# Recognizability of Demographically Altered Computerized Facial Approximations in an Automated Facial Recognition Context for Potential Application in Unidentified Persons Data Repositories

**DOI:** 10.3390/biology12050682

**Published:** 2023-05-04

**Authors:** Connie L. Parks, Keith L. Monson

**Affiliations:** FBI Laboratory Division, 2501 Investigation Parkway, Quantico, VA 22135, USA

**Keywords:** forensic anthropology, facial approximation, facial recognition, unidentified decedents, missing persons

## Abstract

**Simple Summary:**

Forensic anthropologists are tasked with estimating a “biological profile” from bones. This profile includes potentially individualizing characteristics, including sex, age, ancestry, and stature. If only a skull is found and the facial bony structure is intact, its biological profile can be used by a forensic artist or with computer software to construct a facial approximation, which is an informed estimate of how the person may have looked in life. The facial approximation is then released to the public and to other agencies, and facial recognition software may be used to search the approximation against images of missing persons. In case the biological profile is inaccurate due to incomplete or damaged skeletal remains, it is sometimes useful to construct additional facial approximations that are based on alternative sex and ancestry characteristics, especially for use with facial recognition software. This research found that alternative sex facial approximations, when searched against a large gallery of facial photographs, produced higher match scores than did alternative ancestry facial approximations.

**Abstract:**

This study examined the recognizability of demographically altered facial approximations for potential utility in unidentified persons tracking systems. Five computer-generated approximations were generated for each of 26 African male participants using the following demographic parameters: (i) African male (true demographics), (ii) African female, (iii) Caucasian male, (iv) Asian male, and (v) Hispanic male. Overall, 62% of the true demographic facial approximations for the 26 African male participants examined were matched to a corresponding life photo within the top 50 images of a candidate list generated from an automated blind search of an optimally standardized gallery of 6159 photographs. When the African male participants were processed as African females, the identification rate was 50%. In contrast, less congruent identification rates were observed when the African male participants were processed as Caucasian (42%), Asian (35%), and Hispanic (27%) males. The observed results suggest that approximations generated using the opposite sex may be operationally informative if sex is unknown. The performance of approximations generated using alternative ancestry assignments, however, was less congruent with the performance of the true demographic approximation (African male) and may not yield as operationally constructive data as sex-altered approximations.

## 1. Introduction

One of the primary responsibilities of a forensic anthropologist is the examination of human skeletal remains. Based on an analysis of the skeletal remains, the anthropologist develops a biological profile containing estimations of sex, age, ancestry, and stature, and may offer additional assessments of trauma and any potentially individualizing characteristics or anomalies. Currently, forensic anthropologists use several metric and morphological estimation methods based on more than a century of research reporting varying degrees of reliability [1,2,3,4,5,6]. Sex estimation methods, for example, are among the most reliable, with reported accuracy rates of ≈98% when the skeletal remains are in a complete or nearly complete state [7,8]. Conversely, ancestry estimation methods, although still very reliable with reported rates of ≈87% for a complete set of remains, fall short of the accuracy rates of other biological profile estimation methods [5,9,10].

Facial approximation is usually attempted only after other reasonable attempts of identification have failed. The biological profile is essential not only for law enforcement entities charged with resolving cases involving unidentified human skeletal remains, but also for facial approximation professionals similarly tasked with aiding in the resolution of unidentified persons cases. The construction of a useful facial approximation is highly dependent on the knowledge of components of the biological profile, namely sex and ancestry. Anthropological estimation of sex and ancestry, however, is not always possible and depends heavily on the completeness and condition of skeletal remains available for analysis [6,11,12]. Accurate sex estimation, for example, declines to ≈90–92% when only the cranium is examined [7,13]. Genomic and proteomic methods of sex determination are highly successful, even from degraded and archaeological bones [8], and genetic testing offers relatively crude estimations of ethnicity [14] as well as predictions of facial and other phenotypic features [15], but resources do not always permit their use. When a complete set of remains is unavailable, indeterminate estimations and errors may occur, a problematic situation that may falsely exclude individuals from a short list of potential candidates. It is improbable for agencies responsible for unidentified persons cases to invest in the resources necessary to create multiple, alternative demographic facial approximations for each unidentified individual with one or more biological profile parameters ruled as indeterminate (e.g., generating both a male and female approximation for an unidentified individual with no estimated sex).

With the advent of computerized facial approximation applications, this situation has been, at least partially, mitigated. Computerized facial approximation applications offer an alternative to the development of a single facial approximation based solely on the parameters of a biological profile. Computerized facial approximations, once generated using one set of demographic parameters, are generally easily and rapidly regenerated using alternate demographics. The facial approximation application employed in this study, ReFace [16], requires approximately forty-five minutes for the initial preparation and generation of a facial approximation, depending on the computer system employed and level of familiarity with the ReFace application. Using this computer system, regenerating a facial approximation for an individual with alternate sex, for example, requires approximately twenty additional minutes. Generating an approximation with alternative demographics requires only that an operator make selections from an array of demographic options. No additional preparatory steps are required after the initial import of a digital model of a skull and marking 23 cranial landmarks [17]. Further, no specific forensic or anthropology training is required to use ReFace. The ability to rapidly generate more than one approximation for an unidentified individual may offer multiple avenues of investigation to those charged with the task of identification, a situation potentially increasing the number of resolved cases.

Expanding on research previously reported [13,18], this study examines how accurately demographically altered facial approximations are matched with corresponding life photos of the approximated individuals in an automated facial recognition context. The research reported in this paper was conducted in an identification, closed universe, scenario-based mode using a commercial facial recognition system and a real-world operational database [13]. Few studies have reported the use of automated means to search facial approximations against galleries. The results of this study will contribute to the existing literature and demonstrate potential utility for automated facial recognition of computerized facial approximations in a real-world context where portions of the biological profile are indeterminate due to incomplete or damaged skeletal remains or in situations where, even though sex and ancestry have been estimated, additional identification strategies are desired (e.g., estimation of “mixed” ancestry).

## 2. Materials and Methods

The research model and methods employed in this study have been reported [13] and are only briefly discussed here. The reader is encouraged to peruse that study for a detailed explanation of materials and methods. A biometric system may be configured for either 1:1 verification or 1:N identification [19]. The first mode is used to verify or deny an individual based on searching a database of known individuals. This study is designed for use in the second configuration. Its goal is “identification,” defined as including an unknown individual within a group of potentially matching candidates selected by the system from a database (differing from the definition of the term within the forensic community, indicating a positively identified individual). The individual (or approximation) being searched is termed a “probe” and the database is a “gallery”.

The participants (t = 26) for this research were a subset of the original sample of 159 individuals [13]. The participants were males of African descent ranging in age from 18 to 49 years (mean: 31, median: 30), the same African male cohort previously examined [13]. They provided computer tomography (CT) head scans for use in this research that were collected incident to medical diagnostic inquiries. The Institutional Review Boards of collaborating medical institutions and the FBI approved the collection and use of all scans and images employed in this study.

The complete, unaltered image gallery (g) previously constructed was used in this study (described in the appendix to [13]). The gallery consists of high quality, consistently posed, 2D color facial images of Americans ranging in age from 18 to 75 years (average and median age 29 years) with approximately equal representation of both sexes and of three self-identified population groups (African, European, and Hispanic). The gallery images were compiled by random selection from an operational database of 1.4 million color facial images (i.e., mug shots) and included photographs of the 26 participants (g = 6159 images).

**Figure 1:**
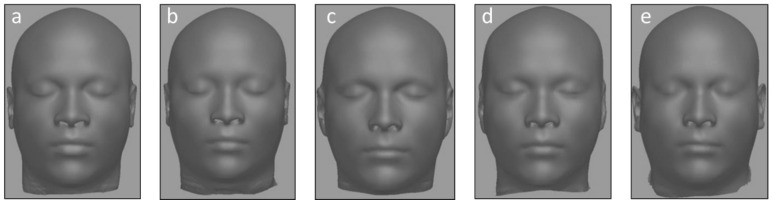
Approximation probe examples for one African male participant. Participant as: (**a**) African male (true demographics); (**b**) African female; (**c**) Caucasian male; (**d**) Asian male; (**e**) Hispanic male.

The probe set consisted of five facial approximations per participant, one generated using each of the following demographic parameters: (a) African male (true demographics), (b) African female, (c) Caucasian male, (d) Asian male, and (e) Hispanic male. Facial approximations were produced using ReFace software (ver. 7.9) installed on a 64-bit, Intel™ Core™ i7 CPU Windows™ system [16]. This application derives a statistically based approximation of a face from either a digital scan of an unidentified skull, or as in this research, from a skull model segmented from a CT or MRI scan. ReFace creates an objective approximation using the skull scan and demographic data of the unknown individual and a reference dataset of human head CT scans. The reference dataset provides bone and tissue depth information of 388 living adult American individuals from four ancestry groups: African, Asian, European, and Hispanic (self-identified). The software generates an “average” approximation based on the weights and ages of the individuals comprising a pertinent subset of the reference database. For example, an African female approximation (probe) would be generated for one of the African male participants by using the weights and ages for the African female subset of the reference database to calculate the average weight and age for this approximation. Typical approximation probes representing one of the participants are shown in Figure 1. Protection of the anonymity of the living participants precludes publication of their images, so the illustrations are computer-generated statistical models that do not reflect the actual visages of any participant.

Each probe was searched against the gallery using NeoFace™ Reveal™ facial recognition software, 2023 (NEC Corp., Irvine, TX, USA) and its candidate rank was recorded. In contrast to the previously reported methodology [13]: (i) only blind searches against the entirety of the gallery were conducted, (ii) no image enhancements were applied, and (iii) the probe set consisted of only the average approximation of each participant. Given that the baseline performance for the facial recognition system employed in this study has been established in two prior research studies [13,18], no internal control was conducted.

We define rank class (R_k_) as the *k* candidates identified by the biometric system as possible matches to the probe. Two overall and cohort performance measures are presented. Candidate list inclusion rate is the fraction of total participants who were identified within a specific rank class. Mean rank reflects the average position of a probe within a rank class. A correct match conveys that a probe is contained within the specified rank class. Fisher’s exact tests were used to analyze performance and paired cohort relationships (e.g., R_50_ inclusion rates of African male approximations vs. African males processed using African females as references). Statistical significance was assessed at *α* = 0.05. Data were analyzed using SPSS 16.0 (Chicago, IL, USA) and Microsoft Excel 2007 (Redmond, WA, USA).

As previously mentioned, no modifications were applied to the final approximations. Instead, the average weight and age approximations for the participants were used as probes. The average approximation represents the initial ReFace output prior to the application of any feature, weight, or age adjustments. A detailed explanation of available ReFace approximation probe types (e.g., average versus heavy weighted), adjustable features (e.g., eye details or skin tone), and image properties can be found in Parks and Monson [13]. It should be noted that the performance metrics reported below for the African male participants’ approximations will differ from those previously reported [13] even though the same African male participants were examined in both studies. While Parks and Monson [13] reported metrics for the top-performing approximations from a set of multiple approximation types, this study examined only the performance of the average approximations with closed eyes. Therefore, the overall performance for the African male cohort reported by Parks and Monson [13] differs from the results presented below.

## 3. Results and Discussion

Although the candidate list inclusion rates (Table 1) between the participants’ true demographic approximations and the rates observed when processed with alternate demographic parameters generally were not statistically different (Table 2), a decline in candidate list inclusion rates was observed with the use of alternative demographics. Overall, 62% of the true demographic facial approximations for the t = 26 African male participants examined were matched to a corresponding life photo within the top 50 (R_50_) images of a candidate list generated from a blind gallery search (g = 6159). In comparison, the most analogous R_50_ candidate list inclusion rate was observed when the African male participants were processed as African females (50%, *p* = 0.58). Less congruent R_50_ candidate list inclusion rates were observed when the African male participants were processed as Caucasian (42%, *p* = 0.27), Asian (35%, *p* = 0.10), potentially reaching statistical significance as Hispanic (27%, *p* = 0.02) males (but not when Bonferroni corrected to *α* = 0.01). A similar pattern of performance was observed irrespective of R_k_ examined.

R_50_ mean ranks observed in this study ranged from 15–18, depending on the alternate demographic parameter applied (Table 3; lower ranks reflect superior performance). These mean ranks are consistent with the upper terminus of the overall blind search R_50_ mean rank range (8–15) reported earlier [13]. The overall blind search R_25_ and R_10_ mean ranks (6–12 and 3–6, respectively) observed in this study were similarly consistent with the mean ranks (6–12 and 3–4, respectively) reported by the authors for corresponding rank classes [13]. While no discernible mean rank performance pattern was apparent across the demographically altered approximations, of note is the contrast between the performance hierarchies of the observed candidate list inclusion rates and mean ranks (Table 1 and Table 3). For example, although the R_50_ candidate list inclusion rate for the African male participants processed as Hispanic males (27%) was the lowest (worst) rate observed, mean ranks for the same Hispanic males were among the best (lowest) mean ranks observed. A similar pattern of performance was observed irrespective of R_k_ examined. However, given that mean rank is not a conventional performance measure for facial recognition technologies, cautious interpretation is advised.

Several U.S. government-sponsored biometric technology evaluations report the applicability and performance of facial recognition systems using photographs as probes in a variety of contexts [20,21,22,23,24,25]. Prabhakar and Bjorn [26] suggest that relatively recent biometric techniques, such as facial recognition, are emerging technologies that are often evaluated with unrealistic performance expectations and are unjustly compared to more mature technologies, e.g., fingerprint analysis. They express that an objective biometric technology, even if not 100% accurate, should perform “satisfactorily” in regard to the specific needs of the target application, i.e., the envisioned use case.

Research examining the performance of computer-generated facial approximations as probes within an objective facial recognition environment is limited. One study generated computerized forensic craniofacial reconstructions of four individuals, then used Picasa^®^ facial recognition software (https://picasa.google.com, accessed on 11 April 2023, ver. 3.9) to search a gallery limited to solely those four individuals, as represented by their high quality photographs, CT-derived skin surface models, and as approximations (i.e., a 1:1 verification exercise) [27]. Two of the photographs were correctly associated with their corresponding approximations. Two additional studies are relevant. The first study found that gender-misclassified images did not increase false match rates of face recognition algorithms [28]. Results of another study attempting automated facial recognition of manually generated facial approximations were disappointing [29]. Photographs of clay approximations of 16 individuals were matched within the top 50 images in only one of 48 match opportunities (one blind and two sex- or ancestry-filtered searches). The gallery consisted of one life photo of each individual and 1800 foils, for a total of 1816 images.

Two objective analyses of ReFace approximations reported candidate list rates. Using methods parallel to the present study, Parks and Monson [18] used facial recognition software to compare 96 ReFace approximations to a large gallery comprised of 96 CT-derived ground truth facial models of those individuals, plus an additional 6159 photographic images. That study showed a high degree of biometric correspondence, with 48% of the ReFace approximations matching to the corresponding ground truth skin surface image at rank R_1_, 85% at R_10_, and 96% at R_25_. Simmons-Ehrhardt et al. [17] employed principal components and linear discriminant analyses to compare 66 central facial region inter-landmark distances between a ReFace approximation and the skin surface of each individual from whose skull the approximation was developed (n = 388). Note that Simmons-Ehrhardt et al. use the phrase match rates rather than the corresponding candidate inclusion rates used by the authors of the current study. Their geometric morphometric approach reports match rates for males and females from four population cohorts in terms of the number of principal components required to achieve the highest match rate for each cohort. The number of components required to achieve “optimal” performance varied widely across cohorts. For example, a match rate of 83% using only four principal components was reported for European males, while 20 principal components were required to achieve a similar match rate (84%) for African females. Three of the four population cohorts examined (African, European, and Hispanic) by Simmons-Ehrhardt et al. were also analyzed in the current study. When taking into account all components for the corresponding three cohorts examined in the current study, Simmons-Ehrhardt et al. match rates ranged from 63% to 88%, with match rates of 73% to 88% (with a varying number of components for each cohort). The match rates of Simmons-Ehrhardt et al. are comparable to the candidate list inclusion rates observed in this study. It must be noted, however, that Simmons-Ehrhardt et al. utilized a gallery of 388 individuals while the present study used a gallery of 6159. Further, Simmons-Ehrhardt et al. examined only linear distance measures of the central face. The current study employed black box facial recognition software that precluded author knowledge of the use of specific facial regions and linear measurements (if any) employed by the proprietary matching algorithms. Although a direct comparison to the study of Simmons-Ehrhardt et al. is difficult given the methodological disparities, comparison is nonetheless instructive and provides preliminary data useful for establishing expected baseline performance measures for ReFace approximations in an objective, automated facial recognition context.

## 4. Conclusions

Using an objective facial recognition system, this research examined the recognizability of ReFace facial approximations generated using alternative demographic parameters. Results suggest that approximations generated using the opposite sex may be operationally informative. In contrast, the performance of approximations generated using alternative ancestry assignments was less congruent with the performance of the true demographic approximation (African male) and may not yield as operationally constructive data as sex-altered approximations. However, given the rapid generation time, creating and processing multiple ReFace approximations with alternative demographic parameters would require minimal resource allocation and may, therefore, serve as an additional tool in a comprehensive identification strategy. It should be noted that the facial recognition system employed in this study is a commercial black box product and the technical aspects of the matching algorithm(s), its specific use of the facial features, and the impact of overlapping variable dependencies are unknown. Due to the unique approach, the black box environment modeled, and the absence of independent assessments of ReFace, the performance metrics observed should not be generalized beyond the scope of this study. Larger studies with more diverse demographics and extended variable analyses are needed in order to more fully evaluate the utility of facial approximations developed with demographic parameters beyond those typically supplied in an anthropologically assessed biological profile.

## Figures and Tables

**Table 1:** Blind gallery search (g = 6159) candidate list inclusion rates by rank class (R_k_).

Participants	R_50_	R_25_	R_10_	R_1_
African males	62%	46%	23%	-
African males as African females	50%	38%	15%	-
African males as Caucasian males	42%	35%	15%	4%
African males as Asian males	35%	27%	12%	8%
African males as Hispanic males	27%	19%	15%	4%

**Table 2:** Fisher’s exact *p*-values by rank class (R_k_) for comparisons of candidate list inclusion rates between participants’ true (African male) and alternate demographic approximations.

Participants	R_50_	R_25_	R_10_	R_1_
African males as African females	0.58	0.78	0.73	1.00
African males as Caucasian males	0.27	0.57	0.73	0.10
African males as Asian males	0.10	0.25	0.47	0.25
African males as Hispanic males	0.02	0.07	0.73	0.10

**Table 3:** Blind gallery search (g = 6159) mean ranks * by rank class (R_k_).

Participants	R_50_	R_25_	R_10_
African males	16	9	6
African males as African females	18	11	5
African males as Caucasian males	15	10	3
African males as Asian males	18	12	6
African males as Hispanic males	15	6	4

* of the probes that ranked within the indicated R_k_.

## Data Availability

Data are unavailable due to privacy restrictions of the informed consent.
